# Correspondence

**Published:** 1880-07

**Authors:** 


### CORRESPONDENCE.

The following letter, though not written for publication, I cannot refrain from giving to the readers of the REGISTER, for it touches upon certain evils that deserve more attention than they have received.

It is humiliating indeed that our colleges in the struggle for numerical success, should sacrifice the chief object for which they were instituted, and thus succeed in fixing reproach where only approbation and honor should be found.

One of two results must occur from the practice referred to in the letter. Either all our colleges must, in popular estimation, be brought down to the low level which some choose to assume, or a line of separation and distinction drawn that will be exceedingly uncomfortable for those on the lower level. What would it avail a physician or any professional man, speaking the English language only, to go to Paris or Vienna, and listen to one or even many courses of lectures in a language of which he only understands a few words; and yet a similar course is pursued by many foreigners who come to this country for a dental education. And why is it? Are such encouraged to come, unprepared as they are? And if so, by whom?

GENEVA, May 30, 1880.

DEAR DOCTOR—Your letter of April 9th reached me just as I was on the point of leaving for Geneva, and I have not had a moment to answer your kind letter. I wish I could write an article on the subject of which I made mention in my letter to Will T. I fear it is a growing evil, and one difficult to overcome. The trouble is to get at the facts in such a form as to pin them in the right place. As long as our colleges vie with each other in trying to show the largest list of graduates, regardless of the quality, we may expect much poor material to be sent out. You know it looks "*distingue*" to have a few graduates of foreign birth, no difference if they do not understand enough of the English language to know what is taught them, or to hardly know enough to find the way to the college after 4 o'clock. No doubt the colleges that are guilty of this nefarious practice think they can send such material back to this country where they will be good enough to practice among a people who are so much behind us in a knowledge of what constitutes good dentistry. But the facts are that this old world is being educated quite rapidly to appreciate American dentistry. Every city on the Continent is represented by some *genuine* American dentist, and their popularity creates a jealousy among the native dentists, and learning that many of their confreres have gone to America and after a few months have returned with a diploma, others in self-defence pick up a few words of English, post off to the land where doctors are made so quick, and return and announce in golden letters that they are American dentists. Diplomas are so common now on the office walls of the native dentists, that those who came abroad a few years ago, so proud of their "sheep-skin" that they had it put in an elegant frame and hung in the parlor, as the one piece of furniture of which they were proud, have taken it down and chucked it among the other rubbish. It is a frequent occurrence that you may enter an office on the Continent with the sign of American Dentist on the door, and if you wanted a tooth extracted and could not speak any other language than English, you could not make the dentist understand what you wanted unless you would make signs as a dumb man. Our patients frequently ask us why we Americans are so far ahead of all other nations in our profession, and frequently make the remark that so many calling themselves American dentists turn out to be mere imitations, and they find often they have been deceived and have not fallen into the hands of the *genuine* article, and they imploringly ask how they may be able to discriminate. To tell them to ascertain if they have a dental diploma is no longer safe, for Ragtag and Bobtail have them. Americans traveling abroad are as often taken in by pretenders as any others. I have known some Americans to go to one calling himself American dentist that speaks English so poorly that when he wants to says he wants to do a big business, he says "wants to do a *pig pusiness*." The standard of our colleges must be elevated, especially in regard to the quality of her foreign graduates, or American dentistry in Europe will become a reproach. I have great hopes of something being accomplished by our little Society, which is composed of the very best of the representatives of American dentistry in Europe, and as president for this year, intend to bring this matter before the Society at our next meeting, the first week in August, at Lucern, Switzerland, and I hope some plan may be devised whereby we may bring the pressure to bear in such a manner on the colleges at home that they may be more careful in regard to the kind and quality of the dentists they make for the foreign field.

We expatriated dentists being deprived of the privilege that we once enjoyed of sitting in our Societies and drinking in the wisdom that fell from the lips of many wise and experienced heads, are somewhat compensated for our loss in having so many very excellent journals to read, which are full of the many good things that are discussed in our Societies. But I often have a yearning desire to be present and see the once familiar faces and grasp the friendly hand. I hope that happy time may not be too far in the future.

I am sorry to learn that my dear old friend Dr. Watt is in such poor health, I hope he may be long spared to the profession of which he has so long been a shining light.

Our heart was made sad at the untimely death of our professions' best friend, S. S. White. Dr. Blount joins me in compliments to you and yours, and all our friends.

